# Spontaneous Reversion of Clinical Conditions Measuring the Risk Profile of the Individual: From Frailty to Mild Cognitive Impairment

**DOI:** 10.3389/fmed.2017.00184

**Published:** 2017-10-30

**Authors:** Marco Canevelli, Giuseppe Bruno, Francesca Remiddi, Carlo Vico, Eleonora Lacorte, Nicola Vanacore, Matteo Cesari

**Affiliations:** ^1^Department of Neurology and Psychiatry, Sapienza University of Rome, Rome, Italy; ^2^National Center for Disease Prevention and Health Promotion, National Institute of Health, Rome, Italy; ^3^Gérontopôle, Centre Hospitalier Universitaire de Toulouse, Toulouse, France; ^4^Université de Toulouse III Paul Sabatier, Toulouse, France

**Keywords:** disability, frailty, mild cognitive impairment, reversion, trajectories, prevention, public health

## Abstract

The number of people living with disabilities worldwide is rapidly growing due to a longer life expectancy and the subsequent increasing burden of chronic diseases. The need of developing and implementing effective strategies aimed at delaying or preventing disability has been repeatedly underlined and is currently the main focus of several health-care policies. In this scenario, a special attention is addressed to the identification of specific clinical conditions measuring the risk profile of the individual of developing an overt disability and other negative outcomes. These risk profiles can indeed become promising targets for developing and implementing preventive interventions. When the disabling cascade is fully established, in fact, the reversing/attenuating the process becomes more challenging. However, the exact nature of these relatively new constructs is not yet sufficiently clear, and several related issues remain poorly explored. In particular, these entities tend to be considered as unequivocally prodromal stages of a future disease, neglecting and underestimating their fluctuations/transitions over time and their potential to clinically improve/revert. This unbalanced judgment did probably contribute to an ambiguous and biased use of these conditions. Considering them as an early stage of an unavoidable future disease, in fact, determined a tendency to start a targeted intervention as if in presence of the disease itself, with the subsequent risk of over-diagnosis and over-treatment. In the present article, we discuss the dynamics underlying the reversion from a clinical at-risk condition to normality and its implications, specifically focusing on the examples of frailty and mild cognitive impairment.

## Introduction

Populations are rapidly aging worldwide as result of a longer life expectancy (1). Such progressive increase in longevity is the sign of major scientific and societal accomplishments (2). However, the longer life expectancy is associated with an increased prevalence of chronic diseases and disabling conditions (3). The number of people living with some form of disability is, in fact, globally growing (4). In this scenario, the identification and implementation of effective strategies aimed at delaying or preventing disability is the main focus of many health-care policies (1, 5).

The early identification and targeting of specific clinical profiles that could potentially serve as targets for preventive actions against disabilities and other negative outcomes has, reasonably and not surprisingly, focused the interest of both research and public health. In fact, once any disabling condition is fully established, the possibilities of functional improvement result drastically reduced.

Several clinical conditions measuring the risk profile of the individual have recently been proposed in different fields of research. Some of these are also gradually acquiring some relevance in the clinical setting (6–9). However, a number of aspects related to these constructs still do not focus enough attention, thus risking to remain poorly explored, to cause misunderstandings, and to be the target of disputable interventions. In particular, these entities are frequently considered as prodromal stages, within a unidirectional pathway toward subsequent disabilities. This approach mistakenly overlooks the possible fluctuations/transitions of the risk profile over time, and do not adequately consider the potential for spontaneous clinical improvement/remission. Such “inverse” trajectories, though commonly observed in routine clinical practice, are often underestimated, thus leading to unbalanced and biased consideration of these conditions.

In the present review, we present and discuss available evidence on the spontaneous reversion to normality from two of the most frequently studied and adopted at-risk conditions, namely frailty and mild cognitive impairment (MCI). Although they have been differently conceived and refer to different functions/domains of the individual, both these constructs have been developed in order to capture and measure the risk of developing poor health-related outcomes. This parallelism allows to address some of the issues potentially arising from such “anticipatory” approach to disabling conditions (i.e., disability and dementia in these cases).

### Reversion of MCI

Mild cognitive impairment is defined as an objective impairment of cognitive abilities that does not affect the subject’s functional independence (9). It is often considered as an intermediate stage in the progression from normal cognitive functioning to dementia (10). To date, the scientific and clinical interest on this construct has mostly been due to its being a condition increasing the risk of developing overt dementia. Subjects with MCI, in fact, show an annual rate of progression to dementia ranging from 5 to 15%, depending on the setting and the considered operational definitions (11). Within this framework, MCI may be considered as a promising clinical condition to identify early signs of a possible progression to dementia and thus design *ad hoc* preventive interventions.

**Figure 1:**
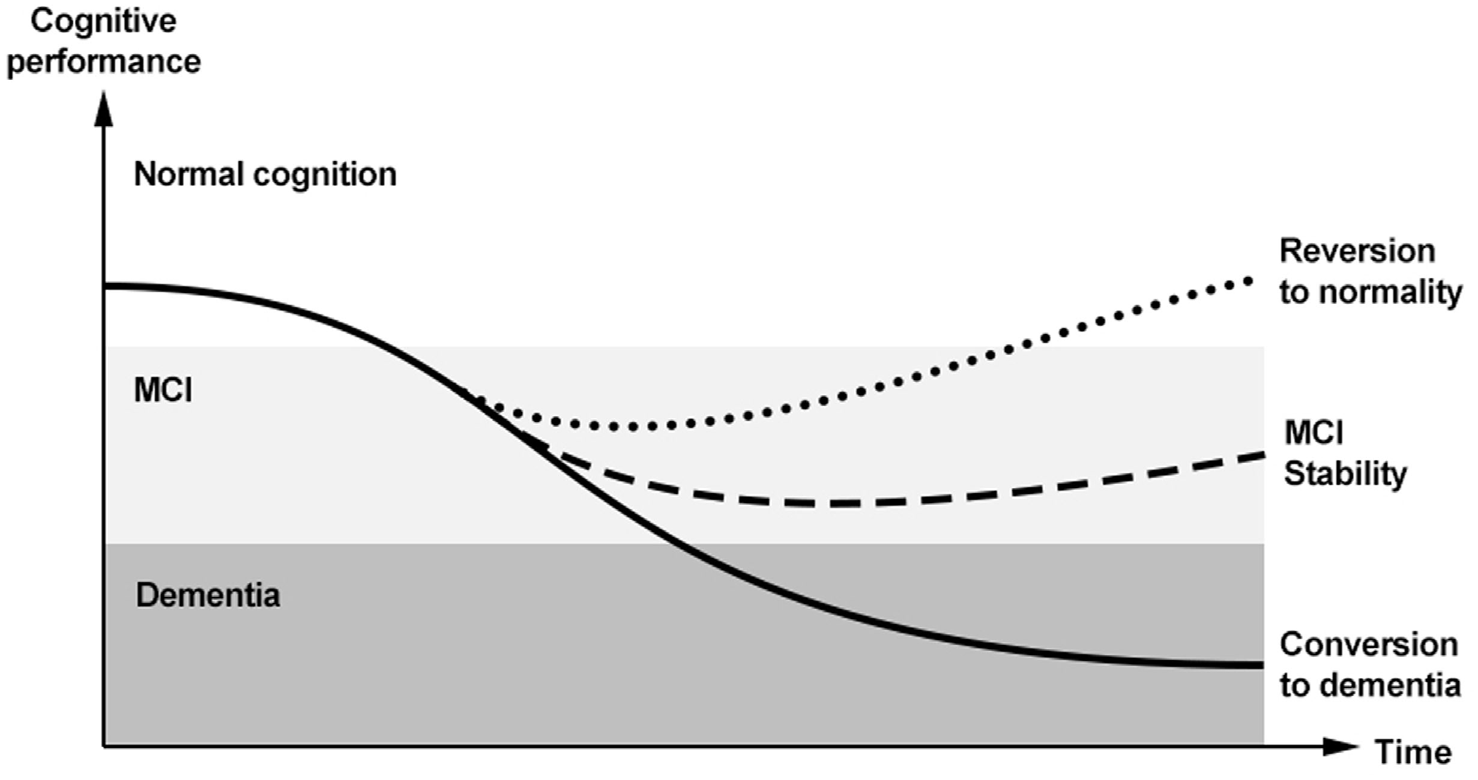
Trajectories of cognitive functioning and potential outcomes of mild cognitive impairment (MCI) in the so-called “dementia continuum.”

However, MCI does not necessarily convert to dementia, but can potentially follow other trajectories over time (12) (Figure 1). The majority of subjects with MCI does not experience a worsening of cognition over time, but tends to remain clinically stable. Population-based studies have actually shown that “stability” might be the most common pattern after a diagnosis of MCI, occurring in 37–67% of the overall cases (12). The limited length of follow-up adopted by available studies on the topic, however, does not allow to draw conclusions on the actual length of this plateau. Anyway, an adequate description of MCI should not omit considering the absence of a conversion to cognitive decline. Moreover, an increasing number of longitudinal data show that a relevant proportion of subjects with MCI may even revert to normal cognition. Two systematic reviews and meta-analyses have recently been carried out to estimate the rate of “reversion” from MCI to normality (13, 14). A first review considered 25 longitudinal studies (published from 1999–2015) enrolling subjects with MCI with a follow-up equal or longer than 2 years (13). An overall 18% (95%CI 14–22%) reversion rate from MCI to normal cognition was observed. In particular, estimates significantly varied according to study setting, with an 8% (95%CI 4–11%) reversion rate in clinical-based studies, and a 25% (95%CI 19–30%) rate in population-based ones. When considering only studies meeting higher quality standards (reported in Table 1), the frequency of reversion further increased to 26%. Consistently, high rates of reversion (31% in the community setting and 14% in the clinical setting) were also documented by a second systematic review, which did not apply restrictions based on the length of follow-up, and only included studies adopting the Mayo Clinic criteria to define amnestic MCI (14).

**Table 1:** Characteristics of available observational studies meeting high quality standards addressing the spontaneous reversion of mild cognitive impairment (MCI).

Reference	Setting	*n*	Sex (%F)	Mean age	Follow-up (years)	MCI definition	Reversion (%)
Grande et al. (15)	C	374	60.2	75.1 ± 6.9	2.7 ± 2.1	IWG	5.6
Roberts et al. (16)	P	534	44.6	na	5.1	IWG	37.6
Sachdev et al. (17)	P	320	51.1	11.5 ± 0.8^a^	1.9 ± 0.1	IWG	20.6
Manly et al. (18)	P	564	68.0	76.5 ± 1.3^a^	5	Mayo Clinic	30.1
Pérès et al. (19)	P	285	57.2	na	2	Mayo Clinic	21.4
Larrieu et al. (20)	P	58	na	na	2	Mayo Clinic	41.4

*Modified from Ref. (13)*.

*C, clinical-based study; P, population-based study; na, not available; IWG, International Working Group criteria for MCI; Mayo, Mayo Clinic criteria for MCI*.

*^a^Weighted mean values*.

*In the present table, we report the six studies of “better quality,” defined using the Quality in Prognostic Studies tool (21), included in a previous meta-analysis on the topic (13)*.

Despite such high rates of reversion, research on the identification of potential factors associated with a favorable trajectory of MCI is still lacking. Evidence from the few available studies indicates that genetic traits (i.e., absence of APOE ε4 alleles), sociodemographic factors (i.e., younger age and higher educational level), clinical features (i.e., greater degree of non-neurological comorbidities), functional independence (i.e., better scores on functional tests), the subtype of MCI (i.e., single-domain non-amnesic), the global cognitive performance (i.e., higher scores at the cognitive tests), and neuroimaging findings (i.e., larger hippocampal volumes) may positively influence the probability of reversion (12, 15, 17, 22).

Another aspect in this field that has not yet been adequately explored is the cognitive stability of subjects reverting from MCI to a normal cognitive function. In the Pittsburgh Cardiovascular Health Study-Cognition Study, a relevant heterogeneity was observed in the clinical course of “reverters.” Some of the participants remained stable within the normal cognition range, but other reconverted to MCI or even progressed to dementia (23). Consistently, another study found that subjects with MCI who reverted to normal cognition were still at an increased risk of developing dementia later in time when compared to cognitively normal subjects (16).

An additional point that needs to be further investigated is whether the adoption of biomarkers reflecting *in vivo* the occurrence of neuropathological modifications may improve the differentiation of the heterogeneous MCI trajectories. Some specific biomarkers might possibly support a better discrimination of MCI cases and help identifying those with a higher probability of progressing to dementia or reverting to normal cognition. Research, however, has been primarily focused on identifying biomarkers associated with a negative outcome (i.e., progression to dementia), largely ignoring those potentially predicting a reversion to normality. It is very likely that these latter cannot be exhaustively found in pathophysiological mechanisms responsible for the onset of the disease, but they may worth the opening of a novel axe of research. In fact, there are reports of clinical reversion to normality among subjects with MCI clearly exhibiting the traditional neuropathological abnormalities (i.e., amyloid deposition) suggestive of an underlying neurodegeneration (24).

### Reversion of Frailty

Frailty is defined as “a medical syndrome with multiple causes and contributors that is characterized by diminished strength, endurance, and reduced physiologic function that increases an individual’s vulnerability for developing increased dependency and/or death” (5). It is also considered as a marker of biological aging and increasingly indicated as a condition of public health interest (25).

To date, frailty has frequently been approached as a pre-disability state (26) and as a condition increasing the risk of adverse health-related outcomes (e.g., falls, functional loss, hospitalization, institutionalization, death) in the elderly (6). Similarly to MCI, much of the interest toward this construct has been due to its ability to predict subsequent negative events. Several studies, however, have proven the dynamic nature of frailty, with frail individuals either worsening or improving over time and showing multiple and bidirectional health “transitions” (6). Again, the potential of frailty for spontaneous clinical remission has, to date, been rarely investigated.

The group of observational studies that have, so far, addressed the spontaneous reversion of frailty are described in Table 2 (27–33). Only articles published on PubMed, from inception to July 2017, or retrieved from the bibliographies of pertaining studies were considered for the present purposes. Available evidence mostly comes from population-based studies enrolling representative samples of community-dwelling older people, with sample sizes ranging from 122 (29) to 15,776 (31) participants, and time spans of observation varying from 1 (29) to 6.4 years (32). One study specifically investigated the transitions of frailty among subjects with cognitive disorders (29). All the studies defined frailty using modified versions of the phenotype proposed by Fried and colleagues (34), which differentiates the specific conditions of robustness, pre-frailty, and frailty. Relevant rates of spontaneous reversion were observed across the available studies, with 13.8 (33) to 44.6% (30) of frail participants reverting to pre-frailty or robustness. One study estimated the possibility of reversion over time using data from three follow-up visits (33). It documented a time-dependent reduction in the probability of favorable transitions. In the overwhelming majority of cases, the most common positive trajectory was toward a pre-frail status rather than to robustness.

**Table 2:** Characteristics of available observational studies addressing the spontaneous reversion of frailty.

Reference	Setting	*n*	Sex (%F)	Mean age	Follow-up (years)	Frailty prevalence (%)	Frailty definition	Reversion (%)
Trevisan et al. (27)	P	3,099	59.7	74.4 ± 7.3	4.4	7.6	mFP	28.2
Jamsen et al. (28)	P	1,705	0.0	76 (median)	2	Na	mFP	Overall transitions to prefailty/robustness: 17.4
		1,366	0.0	78 (median)	2–5	Na		
Chong et al. (29)	C	122	59.4	75.4 ± 7.2	1.0	41.0	mFP	32.0
Lee et al. (30)	P	3,427	43.7	74.0 ± 1.6^a^	2.0	7.9	mFP	44.6
Borrat-Besson et al. (31)	P	15,776	na	na	4.0	6.1	mFP	38.9
Espinoza et al. (32)	P	597	55.1	69.6 ± 3.4	6.4	9.3	mFP	28.8
Gill et al. (33)	P	754	64.6	78.4 ± 5.3	1.5	25.7	mFP	23.0
		679	65.1	79.7 ± 5.2	1.5–3	31.8		17.9
		626	66.3	81.0 ± 5.1	3–4.5	36.7		13.8

*C, clinical-based study; P, population-based study; mFP, modified Frailty Phenotype; na, not available*.

*^a^Weighted mean values*.

Research on the factors associated to or predicting a spontaneous reversion from frailty are still inconclusive. Results from available studies on possible sex differences are conflicting. Some studies, in fact, reported higher reversion rates among women (27, 30), other studies failed to show any significant association between gender and reversion rates, and one study reported that men were more likely to improve (31). Other determinants that were studied as possible predictors of reversion from frailty are younger age (only in women), higher educational levels, living alone, low-to-moderate alcohol consumption, being overweight, and practicing regular physical activity (27, 30, 31). One study found no association between the overall number of medications and burden of anticholinergic drugs, and the progression/regression from frailty over time (28). As for MCI, no consistent data are available describing the long-term trajectories of frail subjects having experienced a spontaneous clinical improvement.

## Discussion

Considering available evidence, reversion should be seen as a quite common outcome of clinical conditions measuring the risk profile of the individual such as MCI and frailty. Knowing that these at-risk profiles have the potential to spontaneously regress, a more balanced and cautious attitude should be adopted when approaching these entities both in clinical and in research settings.

Widening the knowledge on the phenomenon of reversion, within the preventive management of disabling conditions, may have important practical implications. The possibility of identifying those subjects that are more likely to show a positive outcome, in fact, may allow to better allocate health-care resources in the heterogeneous population of aging individuals (35). Moreover, it may prevent possible negative consequences arising from the (mis)diagnosis of a potentially disabling conditions (e.g., discrimination, stigmatization, over-medicalization) (13). Finally, it may improve the design of clinical trials and the interpretation of their results. For example, excluding subjects whose cognitive function or frailty levels are unlikely to decline over time may increase the effect size of potentially effective interventions. As of today, this point seems of crucial interest, considering that nearly 180 RCTs are currently recruiting subjects with frailty and/or MCI worldwide (source: www.clinicaltrials.gov; search updated in August 2017).

Several hypotheses may be proposed to explain the observed remission of the considered at-risk conditions (13). First, it may simply be due to the wrong classification of subjects participating in the studies, with either normal individuals misdiagnosed as frail/MCI at enrollment, or with frail/MCI subjects misclassified as normal at the end of the observation period. According to this hypothesis, the spontaneous remission of the considered conditions might be explained by the weakness of the adopted definitions and diagnostic tools adopted. At the same time, the intrinsic tendency of these entities—that define a risk and not a nosological condition—to fluctuate over time and exhibit unstable courses cannot be ignored. This aspect is strongly related to the multiple factors (e.g., nutritional deficits, affective disorders, cerebrovascular events, sleep disorders, social issues) that can be at the basis of their clinical manifestations. Thus, among the large number of individuals at risk (due to frailty or MCI), there will undoubtedly be a subgroup with the features leading to unavoidable further decline, but there will also be a group, labeled as having a “positive” risk profile, who will not necessarily follow a negative trajectory, and is simply categorized as at risk due to a temporary/reversible condition and/or to a mistake in the evaluation process. The correct definition of these aspects is extremely relevant in terms of public health. The current trend is to extend the boundaries of clinical interventions to at-risk conditions, without adequately considering the possibility of spontaneous reversion, and this is unsustainable in terms of a health economics, because it exponentially increases the number of “individuals to treat.”

Overall, these considerations underline the limits arising from attempting to approach age-related disabling conditions using the traditional medical approach based on a stand-alone disease model. The prevention of clinical conditions cannot meet the same standards and follow the methodologies applied in the treatment of diseases. Defining new conditions to treat does not mean carrying out effective preventive actions. The prevention of age-related conditions requires the adoption of a more naturalistic approach to older subjects, thus should be focused on identifying the trajectories of their functions rather than on a punctual assessment of (arbitrary and categorical) entities. The complexity of health conditions in older age and of age-related disorders might be better approached by adopting measures reflecting the trajectories of personal capacities and abilities, and identifying the interaction between the intrinsic characteristics of each subject and his/her environment (1). This model may better support the personalization of care and the implementation of person-tailored interventions. Special attention should also be devoted to those events or variables that may constitute “switching factors” along the individual’s trajectories, thus positively or negatively modifying the direction of functional and clinical changes over time.

In conclusion, considering their unstable and potentially bidirectional course, MCI, frailty and other similar risk profiles associated with disabling conditions should not be framed into nosological conditions nor considered as prodromal phases of an unavoidable subsequent disease. They should be more adequately approached as the heterogeneous at-risk conditions they were originally designed to be. Such more balanced perspective will allow to reduce the risk of over-diagnosis and over-treatment, and to improve the clinical and research standards in this field. Moreover, the progressive adoption of longitudinal constructs that are more precise at reflecting the complex functioning of aging people and at overcoming the weaknesses of traditional categorical frameworks should be promoted.

## Author Contributions

MCa and MCe designed the study and wrote the manuscript. FR, CV, and EL participated in the review of the literature. GB and NV contributed to the discussion of the available evidence on the topic. All the authors were involved in drafting the manuscript.

## Conflict of Interest Statement

MCe has received honoraria for presentations at scientific meetings and/or research funding from Nestlé and Pfizer. He is involved in the coordination of an Innovative Medicines Initiative-funded project [including partners from the European Federation Pharmaceutical Industries and Associates (Sanofi, Novartis, Servier, GSK, Lilly)]. The other authors have no conflict of interest to declare.
